# Tuberculose multifocale compliquée d’un syndrome d’activation macrophagique: à propos de deux cas

**DOI:** 10.11604/pamj.2019.32.41.17920

**Published:** 2019-01-22

**Authors:** Rajae Azzeddine, Fatimazahra Elyassir, Jamal Eddine Bourkadi

**Affiliations:** 1 Service de Pneumo-phtisiologie, Hôpital Moulay Youssef, CHU Rabat, Maroc

**Keywords:** Tuberculosis, macrophage activation syndrome, antibacillar treatment, immunosuppressants, Tuberculose, syndrome d´activation macrophagique, traitement antibacillaire, immunosuppresseurs

## Abstract

Le syndrome d'activation macrophagique (SAM) est une manifestation rare liée à une stimulation inappropriée des macrophages dans la moelle osseuse et les organes lymphoïdes. Il est défini par des critères cliniques, biologiques et cyto-histologiques. Ce syndrome peut être primaire essentiellement chez l'enfant, ou secondaire à diverses affections: hématologiques, infectieuses ainsi qu'à des maladies auto-immunes variées. Le pronostic est sombre, avec une mortalité de près de 50% toutes causes confondues. Le traitement repose sur le traitement spécifique de l'agent causal du SAM et dans certaines indications, sur l'étoposide, les corticostéroïdes et les immunogobulines qui doivent alors être administrés précocement. Le SAM est rarement décrit en association avec la tuberculose, nous rapportons deux observations de deux patientes, l'une immunodéprimée et l'autre immunocompétente présentant une tuberculose multifocale compliquée d'un syndrome d'activation macrophagique. Nous insistons à travers ce travail sur la difficulté du traitement du syndrome d'activation macrophagique associé à une tuberculose, la nécessité de l'évaluation du rapport bénéfice risque, en raison du risque d'immunosuppression lié à l'utilisation des corticoïdes ou des immunosuppresseurs, avec possible aggravation de la tuberculose.

## Abstract (en)

Macrophage activation syndrome (MAS) is a rare disorder due to an inappropriate stimulation of macrophages in the bone marrow and in the lymphoid organs. It is defined based on clinical and cytohistological criteria as well as on laboratory tests. Primary syndrome can occur mostly in children or it can be secondary to different types of disorders: haematological, infectious diseases as well as different types of autoimmune diseases. Prognosis is poor, with a mortality rate of almost 50% from all causes. Treatment is based on specific treatment dependent upon the causative agent of the MAS and, in some cases, on etoposide, corticosteroids and immunogobulines, which should be administered at an early stage. MAS has been rarely described in association with tuberculosis. We here report two cases: an immunocompromised patient, the first case, an immunocompetent patient with multifocal tuberculosis complicated by macrophage activation syndrome, the second case. This study aims to emphasize on difficulty in treating macrophage activation syndrome associated with tuberculosis, on the need for an evaluation of benefit/risk ratio based on corticosteroid or immunosuppressant-related risk for developing immunosuppression, with possible worsening of tuberculosis.

## Introduction

Le syndrome d'hémophagocytose, aussi appelé syndrome d'activation macrophagique (SAM), ou syndrome d'activation lympho-histiocytaire est une maladie rare mais potentiellement fatale. Le diagnostic repose sur l'association de signes cliniques et biologiques non spécifiques, imposant la recherche cytologique ou histologique d'hémophagocytose et une enquête étiologique exhaustive [1, 2]. Le SAM peut être primitif ou secondaire, les pathologies en causes peuvent être hématologique (lymphome T), tumorale ou infectieuse: virale(en premier lieu EBV), fongique, parasitaire ou bactérienne (plus rarement la tuberculose) [3]. Nous rapportons 2 observations de tuberculose multifocale compliquée d'un syndrome d'activation macrophagique.

## Patient et observation

**Figure 1:**
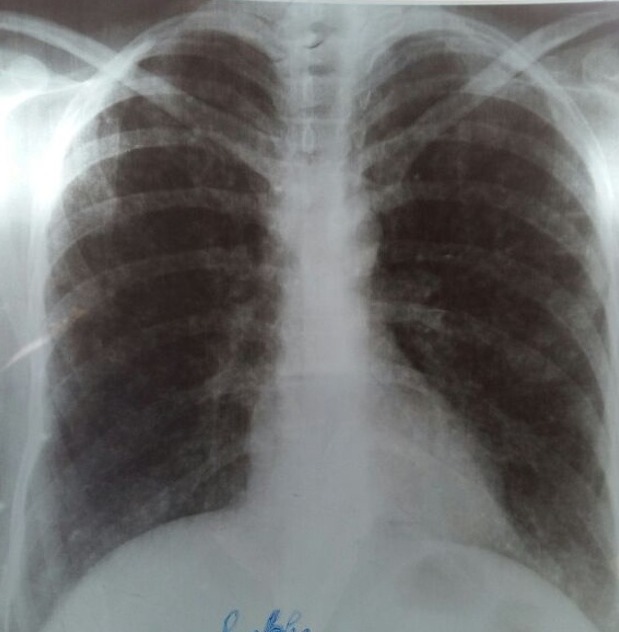
radiographie thoracique de face: opacités micronodulaires diffuses aux deux champs pulmonaire réalisant un aspect de Miliaire (1^ère^ observation)

**1^ère^ observation**: patiente âgée de 43 ans, sans antécédents pathologiques notables, hospitalisée dans notre formation pour une toux sèche associée à une dyspnée d'aggravation progressive, évoluant dans un contexte de fièvre non chiffrée, de sueurs nocturnes et d'altération de l'état général. L'examen pleuropulmonaire était normal, la radiographie thoracique de face objectivait un aspect de miliaire tuberculeuse (Figure 1), la recherche de BK dans les crachats est revenue négative à l´examen direct, alors qu'elle est revenue positive dans le liquide d'aspiration bronchique à la fibroscopie bronchique. Le bilan biologique montrait une pancytopénie: Anémie à 8g/dl normochrome normocytaire régénérative, Leucopénie à 3200 /uL avec une lymphopénie à 380/uL et une thrombopénie à 74000/uL, une hépatite cytolytique: ASAT: 4 fois la normale; ALAT: 2,3 fois la normale, ainsi qu'une choléstase: PAL: 2 fois la normale, et GGT: 3,5 fois la normale, une hyperferrétinémie à 3300 ng/ml; une hypertryglicéridémie à 3,5 g/L, LDH: 1047 U/L, test de coombs direct négatif. Par ailleurs, une sérologie HIV est revenue positive. L'échographie abdominale a mis en évidence une splénomégalie. Devant la présentation clinicobiologique, le diagnostic du syndrome d'activation macrophagique est retenu, Le meddulogramme a montré une moelle riche avec une augmentation de nombre de macrophages et une hémophagocytose, alors que la biopsie ostéomedullaire a mis en évidence un granulome épithélio giganto céllulaire sans nécrose caséeuse. Le diagnostic de la tuberculose multifocale (Tuberculose pulmonaire avec atteinte médullaire) compliquée d'un syndrome d'activation macrophagique chez une patiente immunodéprimée par le VIH est retenu. Un traitement antibacillaire à base de 2RHZE/7RH a été instauré, avec une nette amélioration clinique et biologique (correction de la pancytopénie)

**Figure 2:**
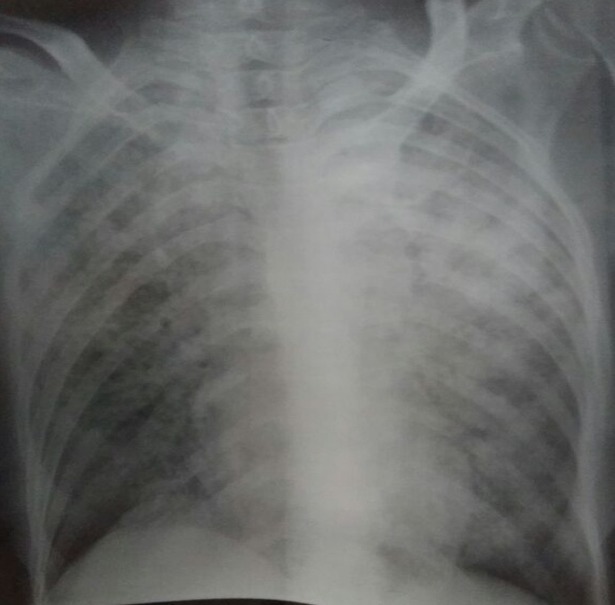
radiographie thoracique de face: opacités micronodulaires diffuses aux deux champs pulmonaire réalisant un aspect de Miliaire (2^ème^ observation)

**2^ème^ observation**: patiente âgée de 45 ans, sans antécédents pathologiques notables, admise initialement au service de gastroentérologie pour des douleurs de l'hypochondre droit associés à des vomissements et une toux sèche évoluant depuis 2 mois dans un contexte de fièvre non chiffrée et d'altération de l'état général avec amaigrissement chiffrée à 12 kg en 2 mois. L´examen pleuropulmonaire était normal, ainsi que la radiographie thoracique. Le bilan biologique montrait une pancytopénie: anémie à 8,4g/dl normochrome normocytaire régénerative, une leucopénie à 2920 /uL avec lymphopénie à 290/uL, et une thrombopénie à 85000/uL, une cytolyse: ASAT à 5 fois la normale, une cholestase PAL: 4fois la normale, GGT: 10fois la normale, bilirubine totale à 16UI, une hyperférritinemie à 11830ng/ml, et une hypertryglicéridémie à 2,8g/l. Le bilan immunologique était négatif ainsi que les sérologies HIV et CMV. L'échographie abdominale réalisée devant la symptomatologie digestive et le bilan biologique perturbé objectivait une hépatosplénomégalie, la ponction biopsie hépatique mettait en évidence un granulome épithélio giganto cellulaire sans nécrose caséeuse à l’étude anatomopathologique. Le diagnostic du syndrome d´activation macrophagique est évoqué devant le tableau clinique et biologique, imposant la réalisation d´un médullogramme qui confirmait le diagnostic en mettant en évidence des signes d'activation macrophagiques. La patiente a reçu une cure d'etoposide. L’évolution était marquée par l´aggravation de la toux ainsi que l´installation d’une dyspnée et une baisse brutale de l'acuité visuelle. La radiographie thoracique montrait alors des opacités micronodulaires diffuses aux deux champs pulmonaires donnant un aspect de miliaire (Figure 2), et la recherche de BK dans les expectorations est revenue positive à l´examen direct. Un fond d'œil a été réalisé devant la baisse de l'acuité visuelle montrant des granulomes choroïdiens avec à la TDM cérébrale des tuberculomes cérébraux. Le diagnostic de tuberculose multifocale (Miliaire tuberculeuse associée à une tuberculose hépatique, oculaire et cérébrale) révélée par un syndrome d'activation macrophagique est retenu. La patiente a été mise sous traitement antibacillaire, avec une bonne amélioration clinique et biologique.

## Discussion

Le SAM est un dysfonctionnement du système immunitaire en rapport avec une hyper activation des macrophages médullaires. La physiopathologie est encore aujourd'hui mal connue: possible hypersécrétion de cytokines (dont le TNF-alpha, IFN-gamma, et IL-6) par des macrophages activés, responsables de manifestations cliniques et biologiques. L'activation de ces macrophages semble due en partie à la sécrétion de cytokines stimulatrices par des lymphocytes T CD8, et Natural killer (NK) activés, ayant perdu leur pouvoir cytotoxique, mais pas leur pouvoir de production de cytokines. Du fait de cette activation inefficace des lymphocytes, l'agent causal de SAM peut persister ainsi que les macrophages. D'où une « boucle autonome » d'activation macrophages-lymphocytes qui augmente de façon disproportionnée. De plus la sécrétion de cytokines inhibitrice pourrait entrainer une dépression de précurseurs granulocytaires et érythropoitiques au niveau de la moelle osseuse [3]. Le diagnostic de SAM repose sur l'association de signes cliniques, biologiques et histologiques ou cytologiques. Les critères diagnostiques ont été récemment redéfinis par un groupe d'expert et sont présentés dans le Tableau 1 [4]. Ils ont été établis pour le diagnostic des formes primaires et sont utilisés par extension pour les formes secondaires [1]. Il faut au moins cinq critères pour retenir le diagnostic de SAM.

**Tableau 1:** critères diagnostiques du syndrome d’activation macrophagique [4]: au moins cinq critères parmi les suivants

**Fièvre**
**Splénomégalie**
**Cytopénies affectant au moins deux lignées:**
Hémoglobine < 9 g/dL
Plaquettes < 100000/mm^3^
Polynucléaires neutrophiles < 1000/mm^3^
**Hypertriglycéridémie et/ou hypofibrinogénémie:**
Triglycérides > 3 mmol/L
Fibrinogène < 1,5 g/L
**Hémophagocytose dans la moelle osseuse, la rate ou les ganglions lymphatiques**
**Pas de néoplasie**
**Activité des cellules *Natural Killer* basse ou nulle** (selon les références du laboratoire local)
**Ferritinémie≥500mg/L**
**Récepteur soluble à l’IL-2≥2400UI/ml**

Cliniquement, on observe habituellement des signes généraux au premier plan avec une fièvre quasi-constante au moment du diagnostic, parfois associée à des frissons, une hépatosplénomégalie pouvant aboutir à une rupture splénique [4]. Des adénopathies périphériques, et des rashs maculopapuleux (Tableau 2) [5]. Les manifestations biologiques portent de façon précoce sur l'hémogramme avec une pancytopénie, et presque toujours une bicytopénie concernant les lignées rouges et plaquettaires. L'anémie est souvent normochrome normocytaire ou microcytaire arégénérative avec des stigmates d'hémolyse (baisse de l'haptoglobine, hyperbilirubinémie libre) et parfois une schizocytose résultant de l'érythrophagocytose médullaire mais aussi sanguine. Le test de Coombs n'est en revanche que rarement positif [6]. Un syndrome mononucléosique avec des macrophages circulants peut être rapporté. On observe de plus une élévation des LDH, de la ferritine et des triglycérides, qui sont des marqueurs utiles au dépistage de la maladie. L'hyperferritinémie (secrétée par les macrophages activés) est présente dans environ 70% des cas et peut atteindre des valeurs jusqu'à 10 fois la normale. Elle représente en outre un bon marqueur d'activité de la maladie et de la réponse thérapeutique et peut être employée comme indicateur pronostique [5, 7] (Tableau 3).

**Tableau 2:** fréquences de signes cliniques du syndrome d’activation macrophagique [5]

Fièvre 70-100 %
Splénomégalie 70-100 %
Hépatomégalie 40-95 %
Adénopathies 15-50 %
Rash cutané 5-65 %
Signes neurologiques 20-50 %

**Tableau 3:** fréquences de signes biologiques du syndrome d’activation macrophagique, d’après [5]

Anémie 90-100 %
Thrombopénie 80-100 %
Neutropénie 60-90 %
Hypertriglycéridémie 60-70 %
Hypofibrinogénémie 65-85 %
Elévation des transaminases 35-90 %
Hyperbilirubinémie 35-75 %
Augmentation des LDH 45-55 %
Hyperferritinémie 55-70 %

**Figure 3:**
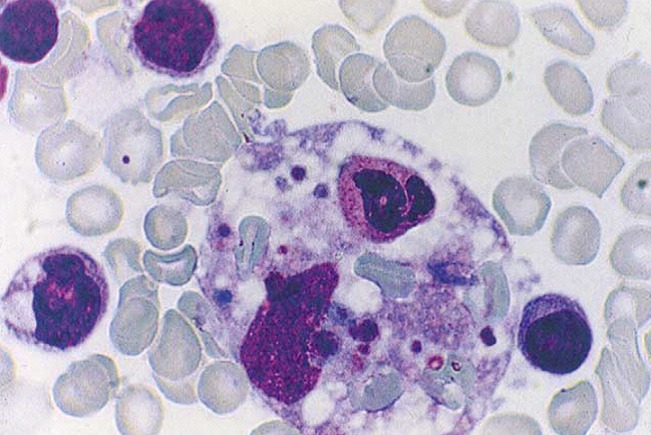
aspect cytologique d’hémophagocytose dans un myélogramme: On aperçoit un macrophage phagocytant un polynucléaire neutrophile et plusieurs hématies

Le myélogramme montre une moelle de richesse cellulaire le plus souvent normale. Les histiocytes hémophages comptent en général pour 2 à 75% des éléments nucléés, sont de morphologie normale et montrent une simple activité phagocytaire. La biopsie ostéomédullaire peut souvent sous-estimer l'hémophagocytose active et semble moins performante que le myélogramme (Figure 3) [2, 8]. L'association du syndrome hémophagocytaire et de la tuberculose reste très rare. Le sujet est discuté depuis plusieurs années : en effet, Cassim *et al*. [9] ont rapporté en 1993 ce qu'ils appelaient une hémophagocytose histiocytaire réactionnelle associée à une tuberculose disséminée ; en 1995 et 1996, Quinquandon *et al*. [10] et Undar *et al*. [11] ont respectivement rapporté deux cas d'hémophagocytose associée à une tuberculose; en 1998, Francois *et al.* ont tenté de rattacher la pancytopénie de la tuberculose à l'hémophagocytose. C'est essentiellement dans les formes de tuberculose extrapulmonaire que le syndrome hémophagocytaire se rencontre. Brastianos *et al*. en 2006, n'ont pu rassembler rétrospectivement que 37 cas dans la littérature, preuve de la rareté de cette association. Parmi ces 37 patients, 29 présentaient une tuberculose extrapulmonaire et 27 avaient une atteinte d'un organe hématopoïétique comme c'est le cas de notre première observation. Dix patients n'avaient pas de localisation hématopoïétique mais présentaient néanmoins un syndrome d'activation macrophagique. Pour trois patients, les données concernant le site de la tuberculose manquent [12]. On peut néanmoins se demander si les cytopénies constatées classiquement dans les cas de tuberculose des organes hématopoïétiques ne seraient pas secondaires en réalité à un mécanisme d'hémophagocytose, cette anomalie cytologique pouvant être méconnue ou peu visible sur les explorations médullaires. Cependant, toutes les localisations hématopoïétiques de tuberculose ne sont pas associées à un syndrome d'activation macrophagique. En effet, la tuberculose splénique, entité rare (68 cas dénombrés dans la littérature depuis une dizaine d'années [13], souvent associée à une atteinte hépatique synchrone, classiquement, ne s'accompagne pas d'hémophagocytose [8].

Le traitement reste encore non consensuel. Dans les formes primaires, un traitement par immunosuppresseur et allogreffe de moelle osseuse est aujourd'hui prescrit. Dans les formes secondaires, le traitement de la cause est essentiel, mais c'est sur le traitement symptomatique de l'hémophagocytose que les avis sont partagés, entre le recours aux corticoïdes, aux immunosuppresseurs ou aux immunoglobulines intraveineuses et l'abstention thérapeutique. Dans les cas associés à une tuberculose, les corticoïdes ou les immunosuppresseurs font craindre le risque d'une aggravation initiale éventuellement fatale de l'infection mycobactérienne. Brastianos *et al*. dans leur série rétrospective, avaient constitué deux bras : un bras avec traitement : subdivisé en un bras avec traitement antituberculeux seul (le cas de notre première observation) et un bras combinant le traitement antituberculeux aux immunosuppresseurs (le cas de notre deuxième observation) et un bras sans aucun traitement. La survie était de 60% dans le bras qui combinait le traitement antituberculeux et les immunosuppresseurs, de 77% dans le bras avec quadrithérapie antituberculeuse seule et de 0% dans le bras sans traitement (diagnostic d'hémophagocytose fait en post-mortem à l'autopsie de neuf patients). Toutefois, le faible effectif et le caractère rétrospectif de l'étude ne permettent pas de tirer de conclusion sur une conduite à tenir consensuelle, du fait de très probables biais de sélection. La question reste donc débattue et nécessite des études de plus grande ampleur. Des études seraient donc nécessaires pour évaluer l'intérêt des traitements immunosuppresseurs et éventuellement des immunoglobulines intraveineuses en association avec le traitement antituberculeux mais la rareté de cette association rend peu probable la réalisation de telles études à grande échelle [8]. Actuellement, la plupart des auteurs s’accordent à préconiser un traitement associant les corticoïdes (au moins 1 mg/kg par jour) à l'étoposide (VP-16) (100 à 150 mg/m^2^) [5, 6, 14, 15]. L'utilisation d'étoposide, composé cytotoxique sélectif de la lignée monocytaire, semble être le facteur déterminant de succès du traitement, et son administration doit être précoce [16]. L'utilisation des immunoglobulines intraveineuses, traitement immunomodulateur dénué d'effet immunosuppresseur, pourrait offrir une alternative séduisante, permettant d'être rapidement efficace sur le syndrome hémophagocytaire, sans prendre le risque d'une flambée de l'infection sous-jacente. Cependant, la place des immunoglobulines dans le traitement de l'hémophagocytose reste à préciser avant que ce traitement ne puisse être proposé dans le contexte d'une tuberculose [12].

Le pronostic du SAM est sombre, avec une mortalité de près de 50 % toutes causes confondues [2], il est essentiellement lié à la maladie associée. L'analyse de quelques grandes séries permet cependant de dégager certains facteurs de mauvais pronostic, indépendamment de l'étiologie. Ainsi, pour Kaito *et al*. [17], le pronostic est plus sombre en cas de thrombopénie inférieure à 100 000/mm^3^ , d'hyperferritinémie supérieure à 500 ng/ml, d'augmentation de la 2- microglobuline plasmatique ou des produits de dégradation de la fibrine (> 10 ,μg/ml) et surtout de cholestase hépatique (bilirubine > 22 μmol/l, phosphatases alcalines > 740 UI/l). La sévérité de la cholestase (et non de la cytolyse hépatique) est également corrélée à un pronostic fatal pour Kerguenec *et al*. [18] dans leur série comprenant 30 patients avec SAM et atteinte hépatique, tout comme l'hypofibrinogénémie et la diminution du facteur V plasmatique. Dans des plus petites séries, l'augmentation d'autres paramètres, non dosés de façon usuelle semble liée à une gravité plus importante: taux plasmatiques de TNFα [19], d'IFNγ, de récepteur soluble à l'IL-2. La mortalité liée au SAM associé à une tuberculose rapportée était de 50 %. Tous les patients qui n'ont reçu aucun traitement antituberculeux sont décédés. Parmi les patients traités, il n'était pas observé d'effet bénéfique de l'adjonction d'un traitement immunomodulateur (stéroïdes essentiellement) aux antituberculeux. Cependant, la grande diversité des cas rapportés, tant sur le plan de l'infection tuberculeuse causale que des traitements reçus, ne permet pas de retenir de conclusions thérapeutiques. Il n'existe actuellement aucune recommandation spécifique pour la prise en charge des SAM dans le cadre d'une tuberculose [1].

## Conclusion

Le syndrome d'activation macrophagique est une pathologie grave, souvent méconnue, pouvant compromettre le pronostic vital, elle peut compliquer diverses maladies infectieuses notamment la tuberculose, néoplasiques ou auto-immunes. La prise en charge du syndrome hémophagocytaire lié à une tuberculose est complexe et non codifiée. L'évaluation du rapport bénéfice risque est difficile, en raison du risque d'immunosuppression lié à l'utilisation des corticoïdes ou des immunosuppresseurs, avec possible aggravation de la tuberculose. A la lumière de nos résultats, nous insistons sur un diagnostic précoce et nous proposons ainsi d'instaurer précocement le traitement antibacillaire dans les SAM compliquant une tuberculose sans associer les immunosuppresseurs pour permettre une meilleure prise en charge, et améliorer le pronostic vital.

## Conflits d’intérêts

Les auteurs ne déclarent aucun conflit d'intérêts.
